# Functional Connectomes in Time Domain from Simulated Neurotransmitter Release Based on Electrocorticograms

**DOI:** 10.3389/fnbeh.2018.00018

**Published:** 2018-02-15

**Authors:** You Zhai, Jian Zhai

**Affiliations:** ^1^The First Affiliated Hospital, Zhejiang University, Hangzhou, China; ^2^Department of Mathematics, Zhejiang University, Hangzhou, China

**Keywords:** functional connectome, connectome in time domain, neurotransmitter release, electrocorticogram, association and habituation

## Abstract

This paper uses a newly defined functional connectome and connectome values calculated in time domain of simulated neurotransmitter release (NTR) from an electrocorticogram (ECoG) to distinguish between conditioned and unconditioned stimuli. The NTR derived from multiple channels releasing one quantum at the same time suggests that one functional connectome occurs across those channels at that time. During the first 600 ms after conditional stimulation, the connectome indexes of the 64-channel NTR trains were sorted from the 8 to 20 Hz band obtained from filtered rabbit ECoGs recorded from the visual cortices. In the small scale visual cortex area, this association was significantly larger than the habituation, even though the trial-to-trail variability of large scale synchrony after conditional stimulation is increased, which is also consistent with the hypothesis that attention decreases coherence of lower frequency within each cortical area. The increased conectome index suggests that the stimuli related to association are able to generate stronger substantial responses in the small scale visual cortex than habituation. That is, besides of the background cortical states as well as attention-related decreases in synchrony of lower frequency, the increased part of neurotransmitters released simultaneously from the pre-synaptic terminals of small scale visual cortex for association is larger than habituation.

## Introduction

Different regions of the brain must communicate with each other to provide the basis for the integration of sensory information, sensory-motor coordination, and many other functions that are critical for learning, memory, perception and the behavior of organisms. Hebb (1949) suggested that this is accomplished by the formation of assemblies of neurons whose synaptic connections are strengthened whenever these cells are activated synchronously. This seminal concept has intrigued investigators, and several methods for calculating synchronization from recorded biological signals that are not spikes have been proposed (see Lachaux et al., 1999; Buzsâki, 2006 and references therein). Current researches on multisensory integration showed that cross-modal (for example, visual-somatosensory) cues that are spatial and temporal coincidence generally enhance the responses of multisensory neurons in cat superior colliculus, whereas those that are spatially or temporally disparate often elicit response depression or fail to be integrated (see Stein et al., 2014 and references therein). In humans and other mammals, a key motif of multisensory integration is the “top-down” control of perception, wherein the primary sensory cortex activity is modulated and controlled by feedback from higher-order regions. In visual cortical areas, many neurons exhibit enhanced responses to attended stimuli and several frontal and parietal cortical regions have been implicated as the sources of top-down modulation signals, especially the dorsolateral prefrontal cortex and frontal eye field (Desimone and Duncan, 1995; Squire et al., 2013; Zhang et al., 2014). In addition to corticocortical projections, frontal eye field also projects to the thalamus and other subcortical circuits that modulate cortical processing (McAlonan et al., 2008; Saalmann et al., 2012). Beyond identifying the signal sources, however, the synaptic circuits mediating top-down modulation and the role of each pathway are largely unknown. This paper aims to use connectome values and simulated neurotransmitter release (NTR) from electrocorticogram (ECoG) data to distinguish between conditioned and unconditioned stimuli. Unlike the usual functional connectomes that are calculated in frequency domain from the phase-locking value (PLV), this newly defined functional connectome is calculated in time domain directly from the NTR train, which is spike-type and has biological meaning. The NTR trains from multiple channels releasing one quantum at the same time suggest that one functional connectome occurs across those channels at that time. The ECoG data used in this paper has been analyzed in Freeman and Barrie (2000) using different methods. In Freeman and Barrie (2000), FFT and principal component analysis were used to derive spatial amplitude modulation patterns within the 20–80 Hz domain and to classify conditioned and unconditioned stimuli. Using the methods in Freeman and Barrie (2000), no distinctive spatial patterns emerged within the 2–20 Hz domain. In this study, sorting NTR trains from ECoG signals revealed a role for the functional connectome related to association or habituation. The striking observation in the present paper is that association learning induces more connectome than habituation. The new functional connectome index can distinguish between association and habituation, while PLV only distinguish between conditional stimulus (CS) onset vs. delay.

Functional connectivity is an observable phenomenon that can be quantified with measures of statistical dependencies, such as correlations of phase and synchrony of aligned individual spikes. The distributed dynamics of hidden neuronal synaptic activity states *x(t)* generally can be described using the following equations:

$$\begin{array}{l} \dot{x}=h(x,\;u,\;\theta )+w\\ y=g\left(x,\;u,\theta \right)+w_{1} \end{array}$$

where *h(x, u*, θ*)* describe the motion of hidden neuronal synaptic activity (Friston, 2011). Since the states *x(t)* are hidden, we need to assess the mapping *g(x, u*,θ*)* from hidden states to observed responses *y(t)*. The parameters θ describe effective connectivity and they control how hidden states in one part of the brain affect the motion of hidden states elsewhere. Furthermore, *u(t)* corresponds to exogenous inputs, *w(t)* and *w*_1_*(t)* are random fluctuations in the motion of hidden states and observations. As noted by Friston (2011), functional connectivity $C=diag{\left(A_{y\;}\right)}^{-\frac{1}{2}}\left(A_{y\;}\right)diag{\left(A_{y\;}\right)}^{\frac{-1}{2}}$ is assessed with the correlation coefficient *A*_*y*_ = 〈*y, y*〉, which are related to effective connectivity θ by the observing equation for *y* and the motion equation for *x*. Generally, the observed responses *y(t)* are a series of signals or a measure of a series of signals. For example, a method, called PLV measures the significance of the phase covariance between two signals. It has been used to examine the role of neural synchronies as a putative mechanism for long-range neural integration during cognitive tasks (Lachaux et al., 1999). Usually functional connectivity *C* is determined by PLV. Given two series of signals *y*_*i*_ *(i* = *1, 2)* and a frequency of interest *f*, this allows the computation of a measure of phase-locking between the components of *y*_*i*_ *(i* = *1,2)* at the frequency *f*. By band-pass filtering each signal between (*f* ± *2* Hz), its convolution with a complex Gabor wavelet *G*(*t, f)* = exp$\left(\frac{-t^{2}}{2\sigma_{t}^{2}}\right)$exp(*j2*π *ft*) centered at frequency f is computed, where σ_*t*_ = 7/*f* (Lachaux et al., 1999). The phase of this convolution ϕ_*i*_*(t, n) (i* = *1, 2)* is extracted for all time-bins *t*, where *n* = *1, 2, 3,…, N* are trial numbers, and for each of the pair of electrodes. The phase locking value (PLV) is then defined at time *t* as the average value:

$$PLVt=\frac{1}{N}\left|\sum_{n=1}^{N}\text{exp }(\text{j}\varphi (\text{t},\text{ n}))\right|$$

where φ (t, n) = ϕ_1_ (t, n) − ϕ_2_ (t, n), j = $\sqrt{-1}$.

As pointed out by Friston (2011), there is a complicated relationship between functional connectivity and the underlying motion of hidden states. In present paper, unlike (Lachaux et al., 1999), a new functional connectome in time domain and connectome values to measure the synchrony of aligned individual neurotransmitter releases are defined. In Freeman and Zhai (2007, 2009) the authors proposed that background cortical states in awake and slow wave sleep could be produced by Poisson-like spike trains propagating through scale-free brain networks. The model used in present paper has been successfully utilized to simulate the power-law and variation in slope of the ECoG PSD in Freeman and Zhai (2007, 2009), helping explain the role of the refractory periods of the spike activity from which the level of background cortical activity is stabilized. This determines the slope of the PSD. This explanation is especially relevant to the change in slope of the PSD of human ECoGs, which averages nearly−2 during awake state and nearly−3 during slow wave sleep. In this sense, the model used in this paper, in addition to the model used in Freeman and Zhai (2007, 2009), are reasonable for measuring hidden neuronal synaptic activity states *x(t)* in hidden state-space. The results here are an advance over previous studies (Freeman and Zhai, 2007, 2009). In this paper, we discover a new functional connectome index related to association and habituation by measuring the hidden neuronal synaptic activity states directly. The NTR derived from multiple channels releasing one quantum at the same time suggests that one functional connectome occurs across those channels at that time. During the first 600 ms after conditional stimulation (CS), the connectome indexes of the 64-channel NTR trains were sorted from the 8 to 20 Hz band obtained from filtered rabbit ECoGs recorded from the visual cortices. The synchrony of aligned individual neurotransmitter releases responding to stimuli is local in space during the initial destabilization (≥600 ms) of primary visual cortex, which is also consistent with previous reports (Cohen and Kohn, 2011; Harris and Thiele, 2011; Salkoff et al., 2015). In the local visual cortex, this association was significantly larger than the habituation, even though the trail-to-trail variability of the synchrony over all visual cortex after CS onset (**Figure 4A** shows that the CS dependent synchronies mainly consist of ~5 channels) is increased comparing with before CS onset (**Figure 9A** shows that the synchronies mainly consist of ≥30 channels), which is also consistent with the hypothesis that attention decreases coherence of lower frequency within each cortical area (Womelsdorf and Fries, 2007; Ruff and Cohen, 2016). The small scale increased conectome index suggests that the stimuli related to association are able to generate stronger substantial responses in the small scale of visual cortex than habituation. That is, besides of the background cortical states as well as attention-related decreases in synchrony of lower frequency, the increased part of neurotransmitters released simultaneously from the pre-synaptic terminals of small scale visual cortex for association is larger than habituation.

In present paper, the mean-field model proposed by Freeman and the second author in Freeman and Zhai (2007, 2009) is extended to characterize the ensemble dynamics of a population of neurons. Simulated neurotransmitter release (NTR) is a mean-field quantity which is the ensemble of neurotransmitter releases of the neurons within the population (an alternative interpretation is the density of neurotransmitter release of a typical neuron within the population). Mean-field models are important because they are parameterized in biological terms. This means their inversion allows one to ask questions that are framed in terms of biological processes, rather than at a purely phenomenological level. The definition of cortical state refers to the dynamics of network activity on a time scale of seconds or more. The defining characteristic of cortical state is the amount of slow fluctuation in the summed activity of a set of neurons (Harris and Thiele, 2011). Synchrony measures the extent to which the timing of individual NTRs is precisely aligned, typically on the timescale of <1 ms. One functional connectome of *k* channels at time t is one synchrony of *k* channels at time *t*, that is, an aligned *k* individual NTRs from *k* channels respectively at time *t*. We call synchrony small scale in case of *k* < *30*, and large scale in case of *k* > *30*.

## Materials and methods

**Figure 1:**
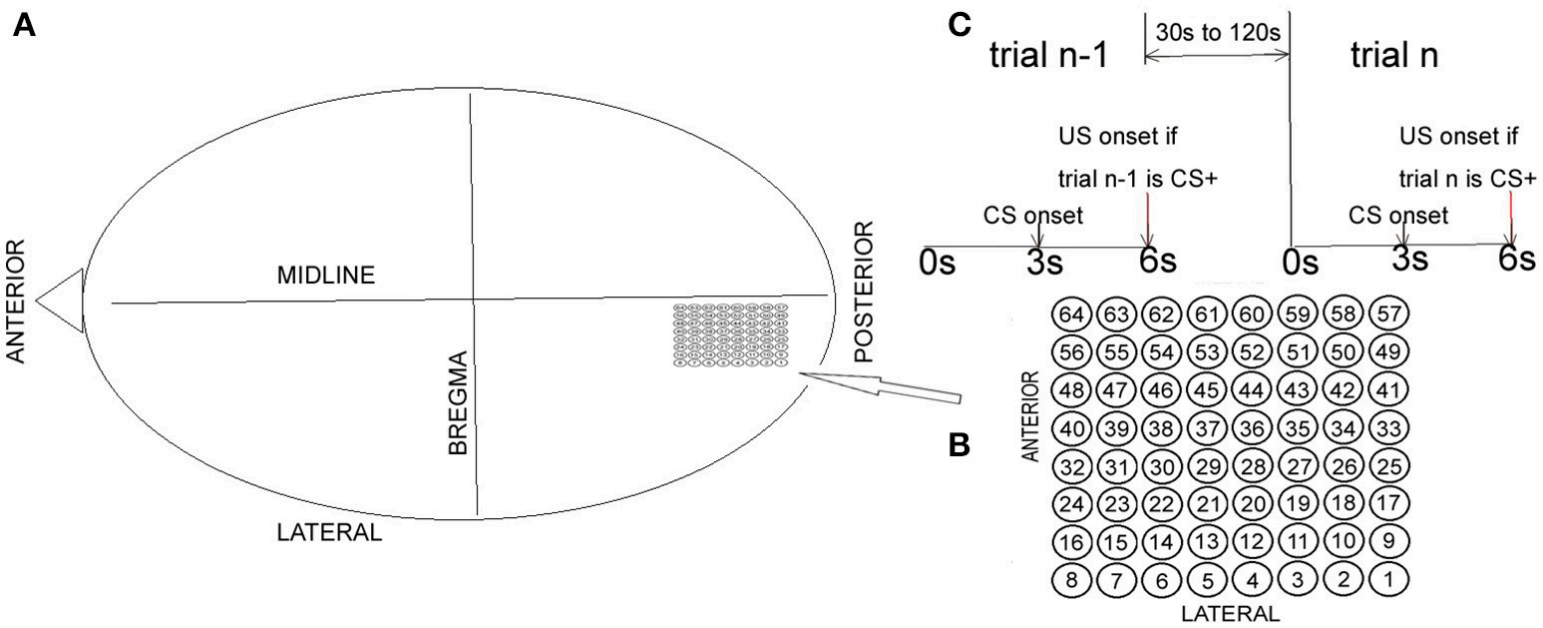
Electrode array and basic summary of the experimental approach. **(A)** The electrode array was chronically implanted onto the epidural surface of the left visual cortex. **(B)** Electrodes and their corresponding numbers. **(C)** Basic summary of the experimental approach.

Data generated by Freeman (see Barrie et al., 1996; Freeman and Barrie, 2000; Freeman, 2005 for details) were used for analysis in the present paper (http://person.zju.edu.cn/old/en/jzhai). In Freeman (2005), two visual, four auditory, and three somatic cortices measurements from a total of nine rabbits were used. The data files used in the present paper included 64 channels of ECoG data recorded from the visual cortex of rabbits (Figure 1). CS+ indicated the type involving reinforcement (association). CS− indicated the type involving no reinforcement (habituation). Two female New Zealand White rabbits were chronically implanted onto the epidural surface of each left cortical hemisphere with an 8 × 8 electrode (0.25 mm diameter stainless steel, epoxy-insulated wire) square array at an average spacing of Δ*d* = 0.79 mm (Barrie et al., 1996). Surgical clips were placed onto the posterolateral aspect of the left cheek for unconditioned stimulus (US) delivery. The restrained animal was placed into an electrically shielded chamber with adequate ventilation and a source of white noise at 72 dB (Barrie et al., 1996). After 1 week of postoperative recovery, each rabbit was familiarized with the experimental setup while being placed in a restraining box to decrease movement artifact in the ECoG recordings. After familiarization, there was one basic experimental paradigm. Each rabbit was classically conditioned to discriminate between two different modality-specific stimuli (see Figure 1C): conditioned stimulus CS+ vs. CS−. The recorded ECoG data for each rabbit consisted of 40 trials with random alternations between CS+ and CS− presentations. The time interval between the two trials was randomized, ranging from 30 and 120 s. Each trial lasted 6 s with an onset of CS at 3 s (duration was 10 ms). During the recording experiment, the rabbit was trained to discriminate between an unreinforced stimulus (CS−) and a reinforced stimulus (CS+) paired to a mild electric shock (unconditioned stimulus). CS− consisted of a weak full-field flash. CS+ consisted of a strong full-field flash paired with a mild electric shock (US), which had sufficient intensity to elicit a skin twitch. The weak or strong flash varied only in luminous intensity (3.6 vs. 2.8 ft.-cd.). During presentation of visual stimuli, each animal was placed in a dark chamber with no background light. The US arrived at the end of the 6 s trial period and it consisted of four to five electrical pulses (1–5 mA) delivered in a window of 10 ms. CS+ was the type with reinforcement (association). CS− was the type with no reinforcement (habituation). At the beginning of the recording experiment, each rabbit learned the task with increased respiration in response to the CS+.

The ECoG was recorded monopolarly with respect to that cranial reference electrode nearest the array and it was amplified by fixed-gain (10K) ISO 4/8 differential amplifiers. Each channel was filtered with single-pole, first-order analog resistance/capacitance filters (6 dB/octave falloff) set at 100 Hz (3 dB point) and 0.1 Hz. Records of 64 12-bit samples multiplexed at 10 μs were recorded at a 2-ms digitizing interval (500 Hz) for 6 s. These records were stored as signed 16-bit integers. The incremental time delay caused by multiplexing of the ECoG was corrected off-line. Bad channels associated with movement artifact or electromyogram were identified by visual editing and replaced off-line by averaging the signals of two vertically or horizontally adjacent channels. These data have been analyzed by Freeman et al. by utilizing power spectral density, in addition to the distance between successive patterns obtained from filtered ECoG signals and their Hilbert transformations (see Barrie et al., 1996; Freeman and Barrie, 2000; Freeman, 2005 for details). Their methods were not involved in the present study. In Freeman (2005) temporal pass bands of 20–80 and 8–40 Hz were adopted. The reason to adopt data from the two visual cortices (40 trails for each rabbit) was the same as in the above papers: the two sets of correct classification values of CS+ vs. CS− showed separation most strongly for the visual cortex and least strongly for the auditory cortex (see the Table 1 of Freeman, 2005).

Here we introduce our method for measuring the synchrony of aligned individual neurotransmitter releases and determining the functional connectome index. The procedure followed four steps.

Step 1. Simulate neurotransmitters using the Poisson process. Neurotransmitters are prepackaged in discrete quantities of a fixed size, called quanta that are identified as the synaptic vesicles universally present at chemical synapses (Stevens, 1993). Some neurotransmitter-filled vesicles (acetylcholine is the transmitter at vertebrate neuromuscular junctions, glutamate is referred to as an excitatory neurotransmitter while GABA is inhibitory) fuse with the membrane of the axon terminus at special release sites to discharge their contents into the synaptic cleft. Communication in nervous system is mediated by action potential-initiated exocytosis of these vesicles. Upon arrival at the postsynaptic membrane, the neurotransmitter molecules induce elementary endplate currents. A synapse has hundreds of release sites. Each site is independent of others and can release either zero or one quantum. A miniature endplate current comprises some 1,000 elementary endplate currents. The potential induced by a miniature endplate current in the postsynaptic membrane is called the MEPP (measuring ~0.5 mV). To depolarize the membrane sufficiently to active an action potential in the soma, some 10 MEPPs need to occur over a short time window (Sakmann, 1992). Based on these biological results for neurons, we simulated the electrical activity of neurons in both forms: NTR trains at pre-synapses and waves of dendritic synaptic current. Here a simulated NTR was regarded as a single-shock electrical stimulation of an afferent pathway. The population activity at the mesoscopic level was modeled by NTRs released by a macro-synapse.

The mathematical description of the Katz theory is a Poisson process. The Poisson process is characterized by the mean rate μ(*t*) (Poisson rate). The Poisson rate is constant or nearly constant in most applications, and μ(*t*) is the average rate at which NTRs occur in a synapse. The μ(*t*) is very low at rest and it then increases dramatically following an action potential. The Poisson process with the Poisson rate μ is denoted by *{x(t): t*≥*0}*, where *x(t)* expresses the total number of NTRs occurring in one synapse. Additionally, *x(t)* starts at *0*, remains unchanged for a holding time *t*_1_ and probability *P(t*_1_ > *t)* = *exp{*−μ*t}*, at time *t*_1_ moves to *1* where it remains for an independent holding time *t*_2_ − *t*_1_ and *P(t*_2_ − *t*_1_ > *t*−*t*_1_*)* = *exp{*−μ*(t* − *t*_1_*) }*. It then moves to *2* at time *t*_2_, and so on. From this model, if an NTR happened in a synapse at time *t*_*j*_, the next NTR will happen in the synapse at time *t*_*j*+1_, with the probability *P(t*_*j*+1_ − *t*_*j*_ > Δ*t)* = *exp{*−μΔ*t}*. The probability that *n* NTRs occur in the same synapse between times *t*_*j*_ and *t*_*j*_ + Δ*t* is considered to be

$$P(n)=\frac{{\left(\mu \Delta t\right)}^{n}\;}{n!}exp\{-\mu \Delta t\;\}.$$

**Figure 2:**
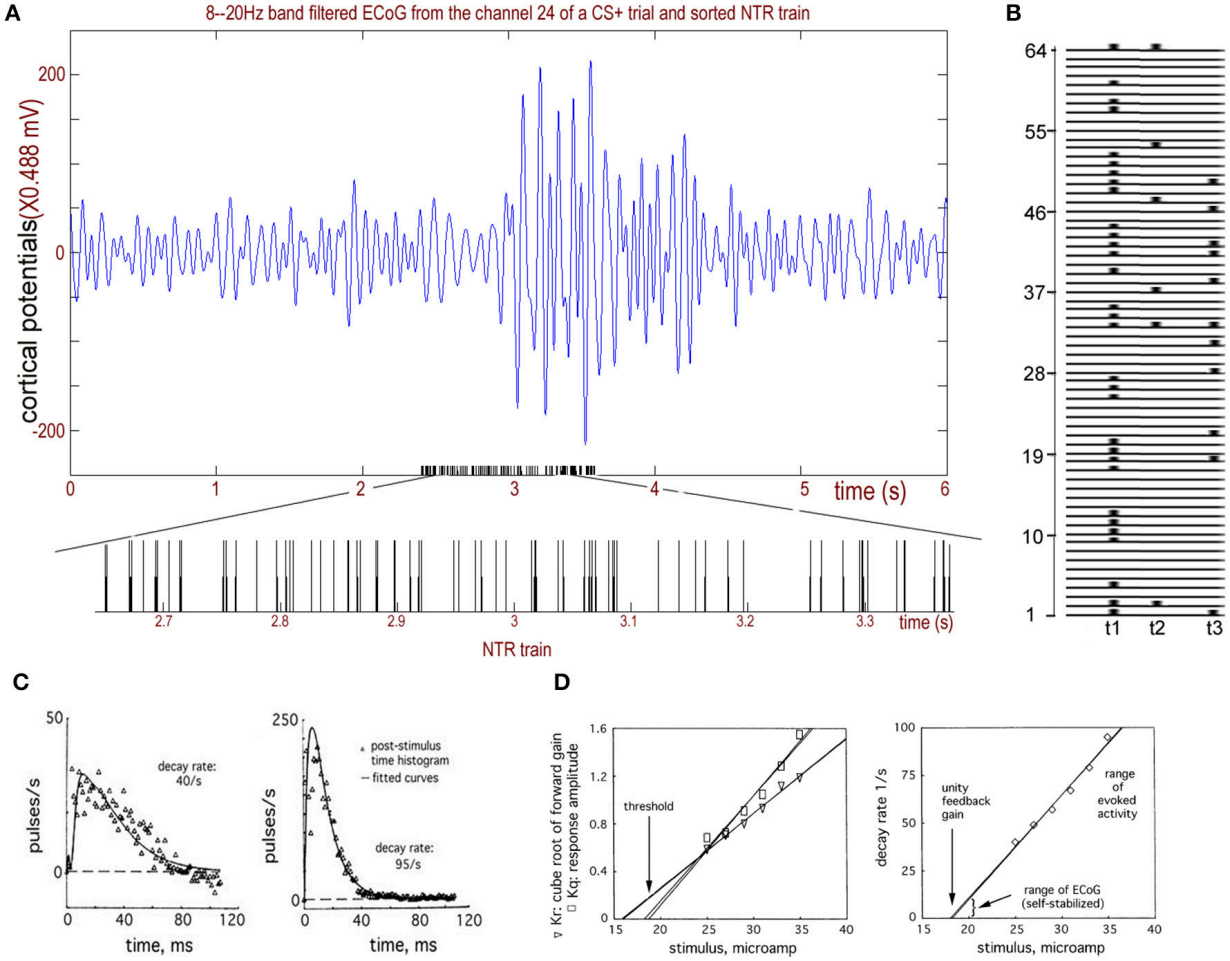
The NTR trains sorted from rabbit ECoGs. **(A)** Upper: Recorded rabbit ECoGs filtered from the 8 to 20 Hz band. Bottom: Sorted NTR trains from the filtered ECoG data. **(B)** Calculating example for T(k) from sorted NTR trains of 64 channels. **(C)** Two examples of the impulse responses of the bulbar periglomerular mutually excitatory population at lowest and highest stimulus intensities give decay rates of 40/s and 90/s, respectively, and rising rates of 500 and 300/s, respectively. **(D)** Left: The relation for the stimulus intensity gives the threshold of the impulse response to an excitatory stimulus; Right: The relation of decay rate to stimulus. Adapted from Freeman and Zhai (2009).

Step 2. Integrate the hidden neuronal synaptic activity states *x(t)* to simulate the ECoG. The population activity at the mesoscopic level was modeled by *p(t)*, which represented extracellular pulse density, and *v(t)*, which represented extracellular wave density and the ECoG. The response *v(t)* to a single-shock electrical stimulation (NTR) of an afferent pathway gave a compound postsynaptic potential with a rapid rise rate, *b*, and a slower decay rate, *a*, that could be approximately fitted with the sum of the following two exponential terms (Figure 2D, see Freeman and Zhai, 2009 for details)

(1) v(t)≈p(t)= ke ∗ (b−a)[exp(−a ∗ t)−exp(−b ∗ t)]

where *ke* was the forward gain parameter and *(b* − *a)* normalized the output magnitude, *p(t)*, with respect to the rate constants in fitting the increase in firing density of population activity above the mean background level after each spike occurrence, with corresponding increase in excitatory synaptic current associated with the increase in pulse density of neural output. The simulated evoked ECoG, *v(t)*, was proportional to *p(t)* by an arbitrary conversion factor between wave density and pulse density (Freeman, 1975). The response to *n* NTRs occurring at time series {*t*_1_, *t*_2_, *t*_3_, …, *t*_*n*_}

$$v(t)=\;\sum_{1\leq i\leq n}ke\;*\;(b-a)[exp(-a\;*\;t_{i})-exp(-b\;*\;t_{i})].$$

**Figure 3:**
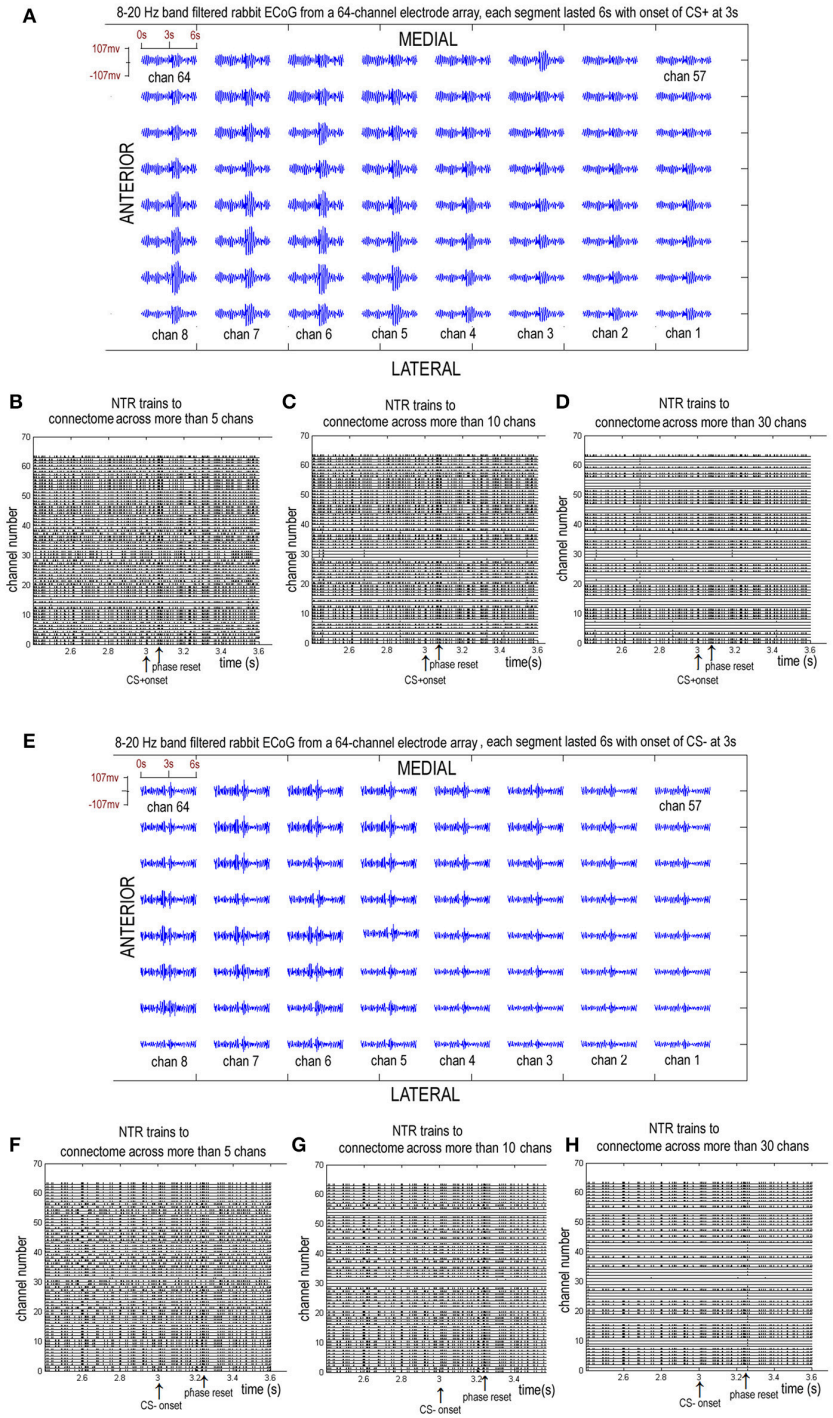
Eight to twenty hertz band filtered rabbit ECoGs from a chronically implanted 64-channel electrode array and the sorted NTR trains corresponding to connectome across more than 5, 10, and 30 channels. **(A–D)** from a CS+ trail. **(E–H)** from a CS− trail.

Step 3. By minimizing the error of these simulated ECoG signals *v(t)* with real ECoG signals *ECoG(t)*, we discovered the best parameters {μ, *ke, a, b*} as well as the simulated series of NTRs, where the signals *ECoG(t)* were derived from the recorded rabbit ECoG signals by solving the inverse problem of conduction equation to reduce the volume conduction (Lachaux et al., 1999). Therefore, we sorted out the NTRs and the hidden neuronal synaptic activity states *x*_*i*_*(t)* from the recorded ECoG signals for each channel *i* = *1, 2, …, 64* (see Figures 2A,B, 3):

$$x_{i}\left(t\right)=\arg\;\min{\;}_{\left\{\mu_{i},\;ke_{i},a_{i},b_{i}\right\}}{\left\|v_{i}-ECoG_{i}\right\|}_{l^{2}},\;i=1,2,\ldots ,64.$$

Step 4. Compute the functional connectome index and connection matrix from the correlation coefficient of the hidden neuronal synaptic activity states *{x*_*i*_*(t)}*_*i* = *1,2, …, 64*_ (see Functional Connectome Index and Results section for details).

Note that Equation (1) is used to simulate the sum of excitatory part “*ke* ^*^ (*b* − *a*)[exp(−*a* ^*^ *t*)]” and inhibitory part “*ke* ^*^ (*b* − *a*)[−exp(−*b* ^*^ *t*)].” Compare this with the work of David and Friston (2003) who showed the excitatory part to be *v*_1_ = *t*(*H*_*e*_/τ_*e*_)*exp*[(−1)*t*/τ_*e*_] and the inhibitory part to be *v*_2_ = −*t*(*H*_*i*_/τ_*i*_)*exp*[(−1)*t*/τ_*i*_]. See Freeman and Zhai (2009) for a serious discussion of the validity of our model used for ECoG data. In particular, simply summing the simulated action potentials cannot simulate the ECoG data because the ECoG is the output of the dendrites that are synaptically driven by action potentials. The simulation by integration must be done by simulating the impulse response of the cortex, which is done by fitting a curve of the cortical responses to a single-shock electrical stimulation (NTR) of an afferent pathway. This impulse response has the forms of a dendritic wave: an averaged evoked potential (AEP) or a fluctuation in the firing density in a post-stimulus time histogram (PSTH, Figure 2C). The impulse response shows a rapid rise and a prolonged return to the background level (Freeman, 1975), owing to the reverberation of firing among the thousands of neurons transmitting and re-transmitting to each other.

### Functional connectome index

Cognitive acts require the integration of numerous functional areas widely distributed over the brain that could be mediated by neuronal groups that produce spatiotemporal patterns of synchronization. These phenomena are called “functional connectomes.” To find a reliable and robust method for measuring such functional connectomes for recorded signals is difficult because the recorded signals are not spikes. Instead, they are local field potential (LFP). Unlike other methods, such as network-based statistic in Zalesky et al. (2010) (which was an approach originally suggested to compute inferential statistics on derived networks), PLV in Lachaux et al. (1999), power spectral density, and the distance between successive patterns obtained from filtered ECoG signals and their Hilbert transformations (Barrie et al., 1996; Freeman and Barrie, 2000; Freeman, 2005), our method is based on the Katz theory. The functional connectome value is obtained directly from NTRs that are spikes and have biological meaning.

Let *k* be an integer between 1 and 64. The NTR trains from the *k* channels releasing one quantum during the same time window *[t*−δ*, t*+δ*]* (for example, δ = *0.2 ms*) suggests that one functional connectome of *k* channels, denoted by *CNT(k,t)*, occurs across those channels at time *t*. Let *T(k)* be the set of the times such that *t*∈*T(k)* if and only if the number of channels having a sorted NTR only at the time window *[t*−δ*, t*+δ*]* is larger than *k*:

T(k)={t:  ∃ CNT(j,t)  with  j≥k}.

That is, at each time window *[t*−δ*, t*+δ*] with t*∈*T(k)*, there are at least *k* channels simultaneously releasing a quantum (NTR). We define the index of functional connectome across more than *k* channels by the following equation:

(2) $$ID(k)=\frac{1}{64}\;\sum_{t\in T(k)}\left\{j:\;\forall \;CNT\left(j,t\right)\text{ with j}\geq \text{k}\right\}$$

For example, in Figure 2B, *t1* and *t2*, in addition to *t3* belong to *T(6)*. Additionally, *t1* and *t3* belong to *T(11), and t1* belongs to *T(31)*. During [t1,t3], the connectome index across more than 5-channels is (31+6+11)/64. For 10-channels, it is (31+11)/64, and for 30-channels, it is 31/64.

A larger index for the connectome implies a larger number or larger scale of functional connectomes. Figure 3 shows the 8–20 Hz band-filtered rabbit ECoG from a chronically implanted 64-channel electrode array and the sorted NTR trains corresponding to connectome across more than *k* = 5, *k* = 10, and *k* = 30 channels.

## Results

### During the first 600 ms after stimulus, the connectome index of the 64-channel NTR trains sorted from 8 to 20 Hz band ECoGs with respect to the association (CS+) is significantly larger than the habituation (CS−) (*p* = 1.1 × 10^−7^, α = 0.05 *t*-test for the connectome across more than 5 channels)

**Figure 4:**
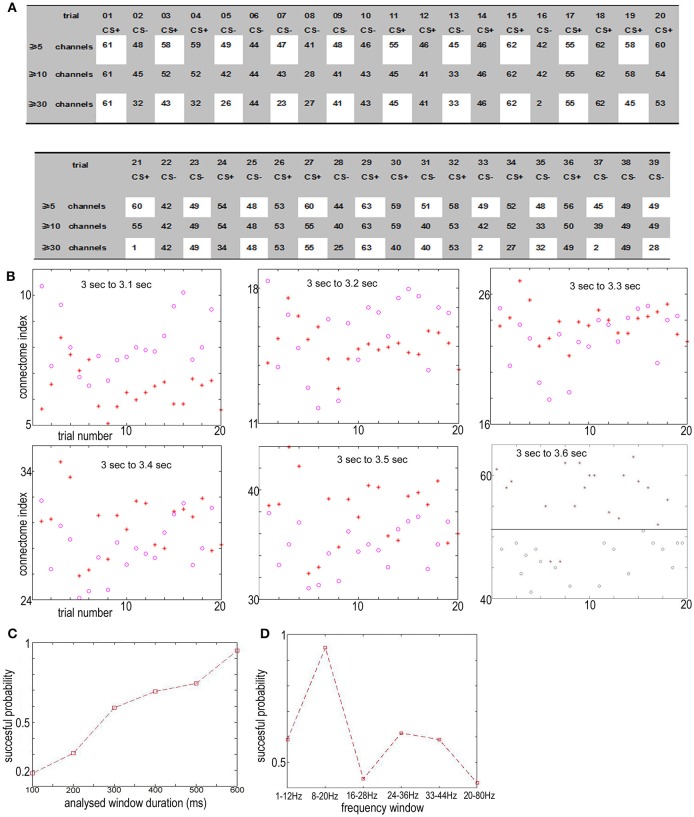
**(A)** The connectome index in the 8–20 Hz band from 3 to 3.6 s (just after CS onset) ranged from trail 01 to trial 39. The three rows show the results of connectome across more than 5, 10, and 30 channels. The connectome index for CS+ was clearly larger than for CS− (*p* = 1.1 × 10^−7^, α = 0.05 *t*-test for the connectome across more than 5 channels). **(B)** Varying the analysis window duration from 100 to 600 ms yielded a window length of 600 ms as optimal for pattern classification by the connectome across more than 5 channels. CS+, red star; CS−, blue circle. **(C)** Successful probability vs. the window duration analyzed for pattern classification for the connectome across more than 5 channels. **(D)** Successful probability vs. frequency windows.

From 3 to 3.6 s, the index for the NTR connectome across more than 5 channels, 10 channels, and 30 channels [ID(5), ID(10), and ID(30)] from trial 01 to trial 39 are shown in Figures 4A, **6A**.

For connectome across more than 5 channels, the threshold to distinguish association learning and habituation equaled 51.5. Only two CS+ trials (trial 11, trial 14, 10.5%) were below the threshold. Meanwhile, for the connectome across more than 10 channels, the threshold to distinguish association learning and habituation equaled 50. Only three CS+ trials (11, 12, 14, 15%) were below the threshold. The results of the Kolmogorov-Smirnov-test confirm that the data: (*ID(5)* for CS+)-(mean value 56.8947) and (*ID(5)* for CS−) – (mean value 46.5263) are independent random samples from normal distributions with mean 0 as well as variance σ = 2 and 1.8 (5% significance level, *p* = 0.0273 and 0.0315), respectively. Furthermore, *p* = 1.1 × 10^−7^, α = 0.05 *t*-test for the connectome across more than 5 channels, and *p* = 0.19, α = 0.2 *t*-test for the connectome across more than 10 channels.

Here only the data between 3 and 3.6 s were used to distinguish between CS+ and CS−. Varying the analysis window duration from 100 to 600 ms yielded a window length of 600 ms as optimal for pattern classification by the connectome across more than 5 channels [*ID(5)*, see Figures 4B,C], which is also consistent with previous reports (Cohen and Kohn, 2011; Harris and Thiele, 2011; Salkoff et al., 2015). As noted by Cohen and Kohn (2011), in the absence of salient changes in the visual scene, animals can only shift their attention approximately once every 400 ms. Even shifts in exogenous attention take 100–200 ms following an abrupt stimulus change. The window length should be larger than the timescale of fluctuations in background cortical state to eliminate the effect of the background cortical state. The synchrony of aligned individual neurotransmitter releases responding to stimuli is local in space during the initial destabilization (600 ms) of a primary visual cortex. In the local visual cortex area, this association was significantly larger than the habituation (*p* = 1.1 × 10^−7^, α = 0.05 *t*-test for the connectome across more than 5 channels), even though the trail-to-trail variability of the synchrony over all visual cortex after CS onset (Figure 4A shows that the CS dependent synchronies mainly consist of ~5 channels) is increased comparing with the indexes before CS onset (**Figure 9A** shows that the synchronies mainly consist of ≥30 channels), which is also consistent with the hypothesis that attention decreases coherence of lower frequency within each cortical area (Womelsdorf and Fries, 2007; Ruff and Cohen, 2016). The increased conectome index suggests that the stimuli related to association are able to generate stronger substantial responses in the small scale visual cortex than habituation. That is, besides of the background cortical states as well as attention-related decreases in synchrony of lower frequency, the increased part of neurotransmitters released simultaneously from the pre-synaptic terminals of small scale visual cortex for association is larger than habituation.

In the basic paradigm, the US (consisted of four to five electrical pulses of 1–5 mA delivered over 10 ms) arrived at the end of the 6 s trial period for CS+ trials. Note that each rabbit was trained on the basic paradigm once per week for a total of three experiments per animal, and the CS−/CS+ contingencies were reversed during the third week with the same animal (the CS+ that was initially paired with the US became the CS− and vice versa). The effect of the flash varying in intensity on connectome index was reduced. Furthermore, in next section, we will show that the connectome index from 0 s to 3 s of trial n was significantly large if the US was onset at the end of trial *n*−1 (see Figure 1C). This suggests that the difference in the connectome indexes is not due to the flash varying in intensity for CS+ and CS− but this is instead due to association and habituation.

Cumulatively, the results showed in Figure 4 suggest that the connectome indexes reflect whether the rabbit learned the association (CS+). On the other hand, Figures 4, **6A** show that for classification of CS+ and CS− from 3 to 3.6 s, the indexes across more than 5 or 10 channels [*ID(5)* or *ID(10)*] are most significant (*p* = 1.1×10^−7^, α = 0.05 *t*-test for 5 channels; *p* = 0.19, α = 0.2 *t*-test for 10 channels). Thus from the definition of connectome index, the large index across more than 5 or 10 channels for CS+ trials from 3 to 3.6 s is due to numerous occurrences of functional connectomes and not the large scale of functional connectomes. The spatiotemporal patterns responding to CS± are local in space during the initial destabilization of a primary receiving area by sensory input, and the spatiotemporal pattern in the 8–20 Hz band from 3 to 3.6 s responding to CS+ trials prefers small scale functional connectomes to large scale of functional connectomes.

**Figure 5:**
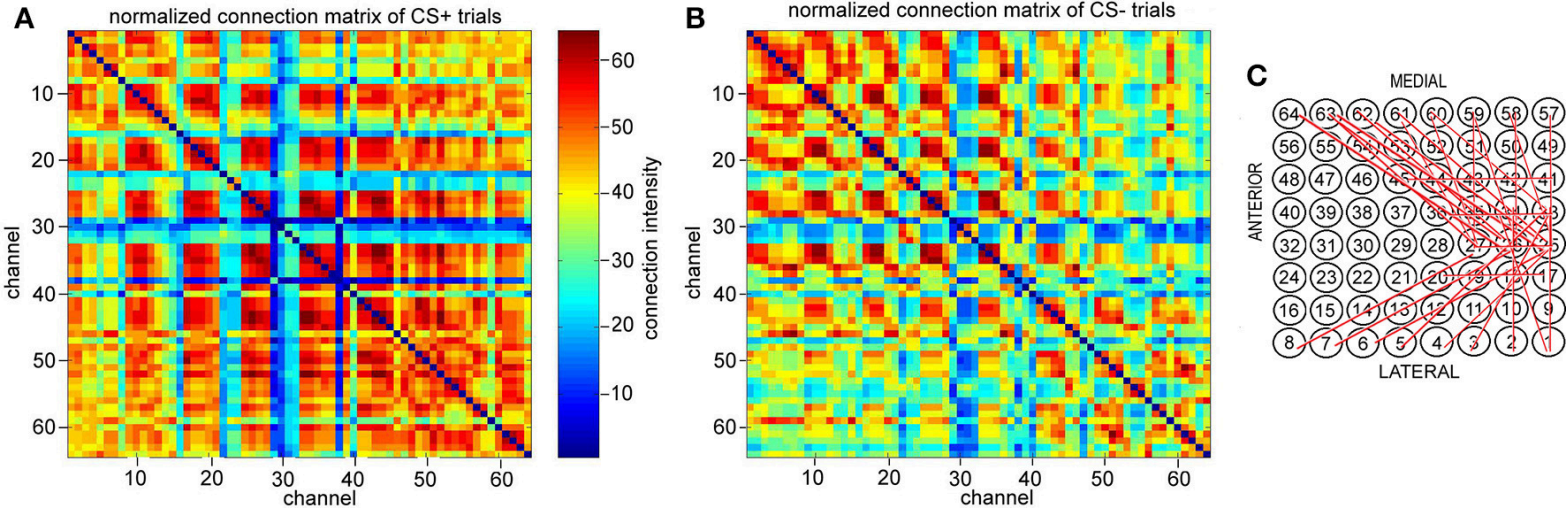
Normalized connection matrix of connectome in 8–20 Hz band from 3 to 3.6 s (just after CS onset) across more than 5 channels. **(A)** CS+ trails. **(B)** CS− trails. There are clearly strong connections from channel 17 to channel 20, channel 25 to channel 27, channel 33 to channel 38, and channel 41 to channel 45 in CS+ trails (*p* = 5 × 10^−7^, α = 0.05 *t*-test). **(C)** The association causes strong local conneting activities across channel 17 to channel 20, channel 25 to channel 27, channel 33 to channel 38, and channel 41 to channel 45 in CS+ trails (active channels/total channels < 28%).

Furthermore, Figure 5 shows which channels were involved in the connectome process. For 1 ≤ *k* ≤ 64, the *k-*connection matrix for CS± trial *n* (*n* = *1,2,3,…,20*) is defined by the following equation:

${\left\{C_{n}^{\pm }\left(i,j;k\right)\right\}}_{1\leq i,j\leq 64}={\left\{\#\left\{t:\exists CNT\left(l,t\right)\text{ such that }l\geq k\text{ and }i,\;j\in \;CNT\left(l,t\right)\right\}\;\right\}}_{1\leq i,j\leq 64},$

where # is the calculating operator of the number of the elements of a set. Let $C_{\max}^{\pm }\left(k\right)=max_{1\leq i,j\leq 64}\underset{1\leq n\leq 20}{\sum }C_{n}^{\pm }\left(i,j;k\right).\;$The normalized connection matrix can be represented as the following:

(3) $${\left\{c_{n}^{\pm }\;(i,j;k)\right\}}_{1\leq i,j\leq 64}=\;\frac{64}{C_{max}^{\pm }\left(k\right)}\;\sum_{1\leq n\leq 20}C_{n}^{\pm }\left(i,j;k\right)$$

Figure 5A is the normalized connection matrix of CS+ trials with *k* = *5*, and Figure 5B is the normalized connection matrix of CS− trials with *k* = *5*. These show the spatiotemporal connecting activities of CS± trials in the 8–20 Hz band from 3 to 3.6 s across more than 5 channels. For CS+ trials, there are clearly strong connecting activities from channel 17 to channel 20, channel 25 to channel 27 and channel 33 to channel 38 as well as channel 41 to channel 45 in CS+ trials (*p* = 5 × 10^−7^, α = 0.05 *t*-test). This suggests that the connectome indexes reflect whether the rabbit learned the association (CS+), and the association causes strong small scale connecting activities across channel 17 to channel 20, channel 25 to channel 27, and channel 33 to channel 38 as well as channel 41 to channel 45 (active channels/total channels<28%). The most significant connections focus on neither medial nor lateral to the area of visual cortex (see also Figures 1A,B).

### Using the connectome indexes of the NTR sorted from the 8 to 20 Hz band of the ECoGs before CS onset or long after CS onset, the association and habituation cannot be distinguished

We compared the results obtained from 3 to 6 s with the same statistics from 0 to 3 s before the CS onset. We found that before the CS onset, the index of the connectome for CS+ trials and CS− trials is completely mixed. A similar phenomenon was also observed as late as 2.4 s after CS onset.

**Figure 6:**
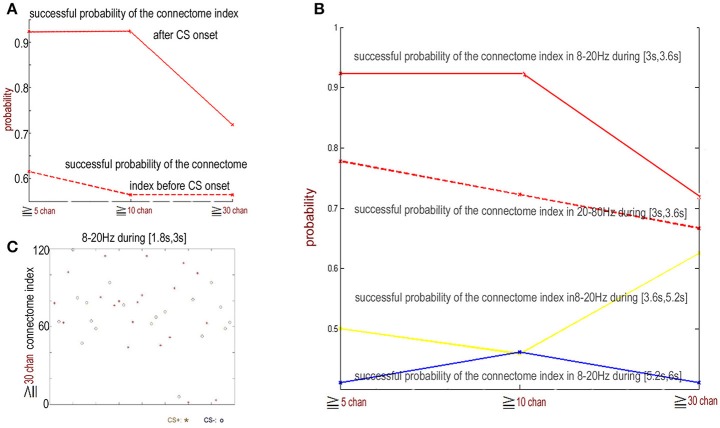
The indexes of the connectome in the 8–20 Hz band from 1.8 to 3 s (just before CS onset) for CS+ and CS− trails are random. **(A)** The successful discriminating probability between CS+ and CS− by the connetome indexes in the 8–20 Hz band from 1.8 to 3 s (just before CS onset) and the connectome in the 8–20 Hz band from 3 to 3.6 s (just after CS onset). **(B)** The successful discriminating probability between CS+ and CS− by the connectome indexes in the 8–20 Hz band from 3 to 3.6 s (red), 3.6 to 5.2 s (yellow), 5.2 to 6 s (blue), and by the connectome indexes in the 20–80 Hz band from 3 to 3.6 s (red dash). **(C)** Indexes of connectome across more than 30 channels in the 8–20 Hz band from 1.8 to 3 s are completely random (successful probability < 0.53). CS+: red star. CS−: blue circle.

Figure 6 shows that from 1.8 to 3 s just before CS+ or CS− stimulus onset, the indexes of the connectome of the NTRs sorted from 8 to 20 Hz band-filtered ECoG data are completely mixed for CS+ and CS− trials on both scales, including the small scale (across more than 5 channels, successful probability < 0.62) and the large scale (across more than 30 channels, successful probability < 0.53). Similar phenomena were observed for time window of 3.6 to 6 s or for the filter band at 20–80 Hz. This suggests that the functional connectome in the 8–20 Hz band for CS+ trials observed in the above subsection only is the short time character of the association learning. The spatiotemporal patterns responding to CS± are local in space (see the above subsection) and time during the initial destabilization of a primary receiving area by sensory input.

**Figure 7:**
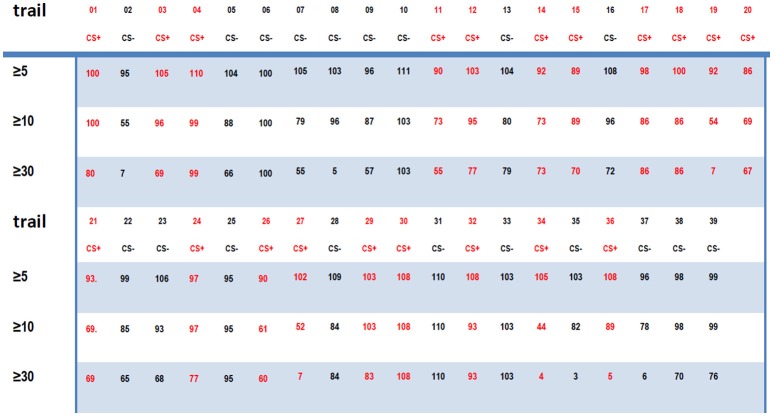
The connectome indexes for the randomized ECoGs.

Furthermore, the connectome indexes for the 64 sequences obtained from the same ECoG data with randomized time order is shown in Figure 7. For 74% of the CS− trails, the connectome indexes of more than 5 channels are larger than 98 [*ID(5)* ≥ *98*]. Meanwhile, for 45% of the CS+ trails, *ID(5)* < *98*. For 60% of the CS+ trails, *ID(10)* < 90. For 52% of the CS− trails, *ID(10)* > 90. For 25% of the CS+ trails, *ID(30)* < 65. For 79% of the CS− trails, *ID(30)* > 65.

**Figure 8:**
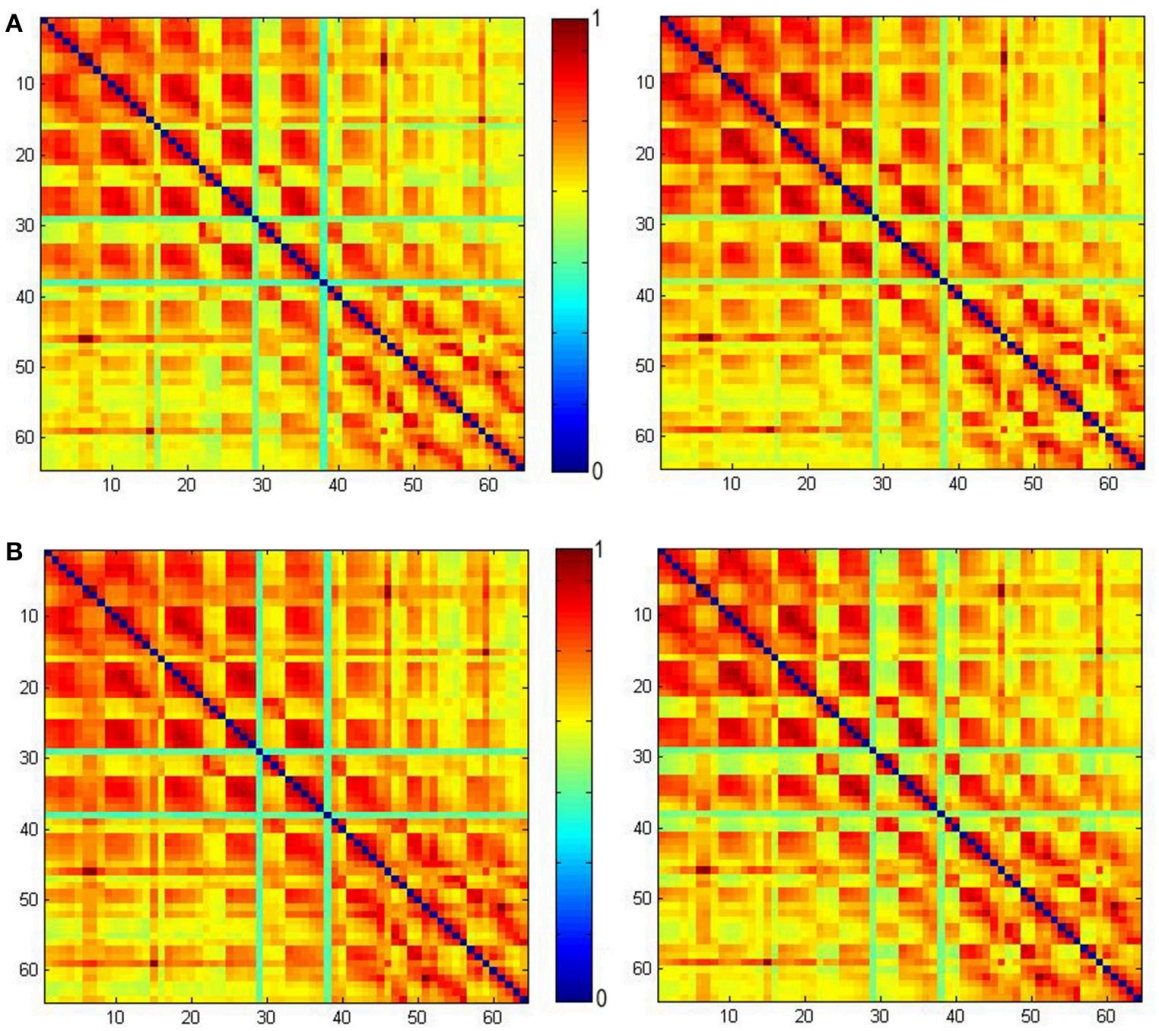
The phase-locking values (PLVs) obtained using the method given in Lachaux et al. (1999) calculated from our ECoG data from 2.4 to 3 s (left) and 3 to 3.6 s (right) filtered by the 8–20 Hz band. PLV for CS+ trails **(A)** and PLV for CS− trails **(B)**.

On the other hand, unlike typical method for defining the functional connectome by calculating phase-locking, the connectome in this paper is determined in time domain using NTRs. Figure 8 shows the PLVs using the method given in Lachaux et al. (1999). This was calculated from our ECoG data from 2.4 to 3 s (the left column) and 3 to 3.6 s (the right column), and filtered using the 8–20 Hz band for CS+ trails (the upper row) and CS− trails (the lower row), respectively. From 2.4 to 3 s (just before the CS onset), the maximum PLV for CS+ trails is 0.0152 the maximum PLV for CS− trails is 0.0153. From 3 to 3.6 s (just after the CS onset), the maximum PLV for CS+ trails is 0.9907, the maximum PLV for CS− trails is 0.9863. Comparing to the connectome index (Figures 4A, 5), there is no significant difference between CS+ trails (Figure 8A) and CS− trails (Figure 8B). The only significant difference before and after the CS onset for both of CS+ and CS− is found. Thus, the PLV in the 8–20 Hz band only represents the CS onset.

### The index of the connectome before CS onset in trial *n* contains the information of US at the end of trial *n*−1 (see Figure 1C)

So far, the following rule pertaining to a trial and the index of the connectome during the trial is as follows: if trial n is CS+ (CS−), then the index during trial n is called the connectome index for CS+ (CS−). On the other hand, it is possible that the index during 0 s to 3 s of trial n may include information on whether a US stimulus was onset at the end of trial *n*−1. We call the connectome index from 0 s to 3 s the former trial connectome index, which is denoted by ***FID(k)***. The former trial connectome index of trial *n* is CS+ (CS−) provided that trial n−1 is CS+ (CS−).

**Figure 9:**
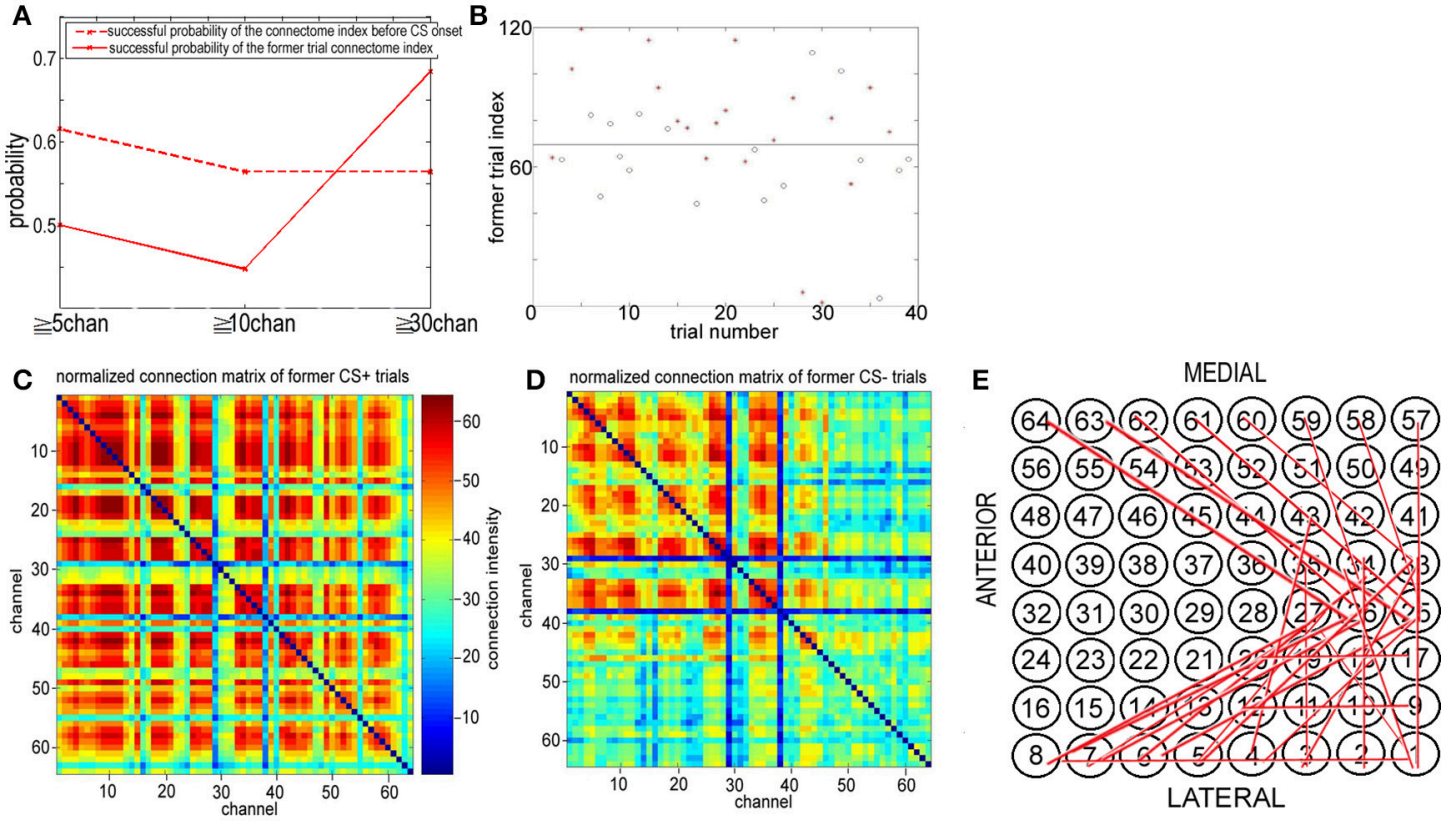
Former trail connectome index. **(A)** The successful discriminating probability between CS+ and CS− trails based upon the connectome index before CS onset and the former trail index of the connectome in the 8–20 Hz band from 1.8 to 3 s (just before the next CS onset). **(B)** Former trail index of the connectome in the 8–20 Hz band from 1.8 to 3 s across more than 30 channels. Former trail CS+: red star. Former trail CS−: blue circle. **(C,D)** Normalized connection matrix of former trail connectome in the 8–20 Hz band from 1.8 to 3 s across more than 30 channels. There is clearly large scale strong connecting activity after US on set (*p* = 3 ×10^−88^, α = 0.05 *t*-test). **(E)** The most significant connections focus on channel 1 to channel 12, corresponding to the lateral area of the visual cortex.

Figure 9 shows the new rule applied to the same data in Figure 6C. In contrast, in the former subsection the old rule fails to distinguish between CS+ and CS−. The successful discriminating probability between CS+ and CS− by the former trial index of the connectome in the 8–20 Hz band from 1.8 to 3 s (just before the next CS onset) is significantly larger than the successful discriminating probability using the old rule (the successful probability of the index of the connectome more than 30 channels: the probability for the new rule ≈0.7, the probability for the old rule < 0.53). This clearly shows that if a US stimulus is onset at the end of trial *n*−1, about 70% *FID(30)* of the 8–20 Hz indexes of the connectome across more than 30 channels from 1.8 to 3 s of trial n is larger than 70, and if there is no US stimulus at the end of trial n-1, about 70% *FID(30)* < 70.

Cumulatively, the former trial connectome index across more than 30 channels [*FID(30)*] for association learning is significantly larger than habituation, and the connectome indexes from 0 to 3 s of the trial n do include information on whether a US stimulus was onset at the end of trial *n*−1, even though the time interval between the two trials is larger than 30 to 120 s (see Figure 1C). This suggests that the spatiotemporal pattern responding to US onset persists in time. Note that US stimuli were delivered to the posterolateral aspect of the left cheek by surgical clips. The large scale synchronies of visual cortex responding to US onset must be induced by communication between visual cortex and other cortical areas. Figures 9C,D show the normalized connection matrix of former CS+ trials and former CS− trials, respectively, with *k* = 30. The connection matrices of former CS± trials were obtained in the 8–20 Hz band from 1.8 to 3 s across more than 30 channels. In former CS+ trials, there is clearly large-scale strong connecting activity involving almost all channels (active channels in former CS+/active channels in former CS−>2), which suggests that the connectome indexes reflect whether the rabbit learned the association (CS+). Furthermore, the association causes strong large-scale connecting activities. The most significant connections focus on channel 1 to channel 12, which correspond to the lateral area of the visual cortex (Figures 1A,B, 9E).

## Discussion

A new functional connectome definition and computing method using simulated NTR trains from ECoG are used to estimate the connectome value and distinguish between conditioned vs. unconditioned stimuli. Synchrony can be calculated directly from NTR trains, which are spike-type and have biological relevance. The small scale connectome indexes of the NTR among 64 channels with respect to association learning are significantly larger than that for habituation. The results observed in the present paper suggest that association learning locks the 8–20 Hz band in different space and time scales after CS+ or US stimulus onset. This reflects the difference in information processes between association and habituation, as well as the different periods of association learning.

For classification of CS+ and CS− during the time interval [3, 3.6 s] in each trial, the connectome indexes across more than 5 or 10 channels [*ID(5) or ID(10)*] of NTR trains sorted from the 8–20 Hz band for the ECoG, are most significant. This suggests that the spatiotemporal patterns responding to CS± are small scale in space during the initial destabilization of a primary receiving area by sensory input. The connection matrix in Figure 5 shows that the most significant connections are focused on neither medial nor lateral area of the visual cortex.

On the other hand, Figures 9A,B shows that the indexes of the large scale connectomes of NTR from 8–20 Hz before CS onset in trial n reflect the information of US at the end of trial *n*−1. If trial *n*−1 is the CS+ type, about 70% *FID(30)* of the indexes of the connectomes across more than 30 channels of NTRs from 8–20 Hz from 1.8 to 3 s of trial n are larger than the threshold. Otherwise, there is no US at the end of trial *n*−1, about 70% of the indexes of the connectomes across more than 30 channels from 1.8 to 3 s of trial n are less than the threshold. Therefore, the former trial connectome index across more than 30 channels for association learning is significantly larger than that for habituation, and the connectome indexes from 0 to 3 s of trial n do include the information on whether a US was onset at the end of trial *n*−1, even though the time interval between two trials is larger than 30 to 120 s (see Figure 1C). This suggests that the large scale synchronies of visual cortex responding to US onset persist in time and induced by communication between visual cortex and other cortical areas. Figures 9C,D shows the connection matrix of former CS+ trials and former CS− trials, respectively. In former CS+ trials, there is clearly large-scale strong connecting activity involving almost all channels (active channels in former CS+/active channels in former CS−>2), which suggests again that the connectome indexes reflect whether the rabbit learned the association (CS+). The most significant connections focus on channel 1 to channel 12, consisting of the lateral area of the visual cortex (Figures 1, 9E). We also found the *t*-test result (*p* = 0.12, α = 0.15) for the successful discriminating between CS+ and CS− by the former trial index of the connectomes across more than 30 channels of NTRs from 8 to 20 Hz from 1.8 to 3 s. The *t*-test result coincides with the successful probability 0.7.

Unlike typical methods for defining the functional connectome by calculating phase-locking, the connectome in this paper is determined in time domain using NTRs. Figure 8 shows the PLVs using the method given in Lachaux et al. (1999). These are calculated from our ECoG data from 2.4 to 3 s (the left column) and 3 to 3.6 s (the right column)filtered by the 8–20 Hz band for CS+ trails (the upper row) and CS− trails (the lower row), respectively. From 2.4 to 3 s (just before the CS onset), the maximum PLV for CS+ trails is 0.0152, and the maximum PLV for CS− trails is 0.0153. From 3 to 3.6 s (just after the CS onset), the maximum PLV for CS+ trails is 0.9907, and the maximum PLV for CS− trails is 0.9863. Comparing to the connectome index (Figures 4A, 5), there is no significant difference between CS+ trails (Figure 8A) and CS− trails (Figure 8B). The only significant difference before and after the CS onset for both of CS+ and CS− is found. Thus, the PLV in the 8–20 Hz band only refers to the CS onset.

## Significance statement

Unlike the typical functional connectome that is calculated in frequency domain from phase-synchronization, a newly defined functional connectome is developed in time domain. The connectome values can be calculated directly from the NTR, which is spike-type and has biological significance. The new functional connectome index can distinguish between association and habituation, while PLVs only distinguish between the conditional stimulus onset and termination.

## Ethics statement

Data were provided by W. Freeman (UC Berkeley). All procedures were conducted according to protocols approved by the University of California at Berkeley Animal Care and Use Committee with veterinary supervision by the Office of Laboratory Animal Care.

## Author contributions

All authors listed have made a substantial, direct, and intellectual contribution to the work, and approved it for publication.

### Conflict of interest statement

The authors declare that the research was conducted in the absence of any commercial or financial relationships that could be construed as a potential conflict of interest.
